# MiR-223-3p in Cardiovascular Diseases: A Biomarker and Potential Therapeutic Target

**DOI:** 10.3389/fcvm.2020.610561

**Published:** 2021-01-20

**Authors:** Meng-Wan Zhang, Yun-Jie Shen, Jing Shi, Jian-Guang Yu

**Affiliations:** Department of Pharmacy, Shanghai Chest Hospital, Shanghai Jiao Tong University, Shanghai, China

**Keywords:** cardiovascular diseases, miR-223-3p, signaling pathway, cardiomyocyte, biomarker

## Abstract

Cardiovascular diseases, involving vasculopathy, cardiac dysfunction, or circulatory disturbance, have become the major cause of death globally and brought heavy social burdens. The complexity and diversity of the pathogenic factors add difficulties to diagnosis and treatment, as well as lead to poor prognosis of these diseases. MicroRNAs are short non-coding RNAs to modulate gene expression through directly binding to the 3′-untranslated regions of mRNAs of target genes and thereby to downregulate the protein levels post-transcriptionally. The multiple regulatory effects of microRNAs have been investigated extensively in cardiovascular diseases. MiR-223-3p, expressed in multiple cells such as macrophages, platelets, hepatocytes, and cardiomyocytes to modulate their cellular activities through targeting a variety of genes, is involved in the pathological progression of many cardiovascular diseases. It participates in regulation of several crucial signaling pathways such as phosphatidylinositol 3-kinase/protein kinase B, insulin-like growth factor 1, nuclear factor kappa B, mitogen-activated protein kinase, NOD-like receptor family pyrin domain containing 3 inflammasome, and ribosomal protein S6 kinase B1/hypoxia inducible factor 1 α pathways to affect cell proliferation, migration, apoptosis, hypertrophy, and polarization, as well as electrophysiology, resulting in dysfunction of cardiovascular system. Here, in this review, we will discuss the role of miR-223-3p in cardiovascular diseases, involving its verified targets, influenced signaling pathways, and regulation of cell function. In addition, the potential of miR-223-3p as therapeutic target and biomarker for diagnosis and prediction of cardiovascular diseases will be further discussed, providing clues for clinicians.

## Introduction

Cardiovascular diseases (CVDs), as the first leading cause of death from non-communicable diseases worldwide, include atherosclerosis, coronary artery disease, heart failure, hypertension, arrhythmia, etc (1–3). Deaths from CVDs are increasing in recent years, mainly resulting from the global aging and population growth (2–5). The driving causes of cardiovascular system dysfunction refer to multiple factors such as aging, hyperlipidemia or atherosclerosis, hypertension, inflammatory response or infection, and genetic influence. Although medical advances in diagnosis and treatments of CVDs, as well as methods to improve prognosis of these diseases, have made benefits for patients, the burden of CVDs remains huge in all regions of the world, as one of the vital obstacles of sustainable development of human (2, 3, 6). Current techniques and drugs to treat CVDs are still limited and the diagnostic methods are often complex and invasive, therefore convenient and effective biomarkers and therapeutic targets for CVDs are urgently needed.

MicroRNAs (miRNAs/miRs), as a kind of non-coding RNA, have been proved to participate in multiple cardiovascular disorders and varieties of pathological processes of CVDs, such as atherosclerosis, coronary artery disease, heart failure, cardiac hypertrophy, cardiac remodeling, arrhythmia, and myocardium ischemia (7–10). For example, miR-208, which is cardiac-specifically expressed, is the first miRNA to be found affecting cardiac hypertrophy. MiRNAs have been increasingly recognized as the potential therapeutic target for CVDs and the miRNA-based therapeutic approaches to perform in clinical applications are challenging but promising (11). MiRNAs are synthesized firstly in the nucleus through transcribing the genes from DNA to primary miRNAs and then processing them to precursor miRNAs (12–15). The precursor miRNAs, which are transferred from nucleus to cytoplasm, are cleaved to become the mature miRNAs duplex (16). In most cases, one of two strands (5p/3p) of mature miRNAs duplex are degraded or less active and is called the passenger strand, while the reserved strand is often identified as the guide strand to form miRNA-induced silencing complex and executes the active regulatory function, although sometimes the passenger strand is also functional (17–19). The canonical mechanisms underlying the post-transcriptional gene silencing by miRNAs are directly binding to the 3′-untranslated regions of the target mRNAs, which lead to the degradation of mRNAs and the reduction of protein levels (20, 21). Besides intracellular gene regulation, miRNAs can be also transported by microparticles, exosomes, or binding to proteins in body fluids, as well as in the free form in blood, to play a part in intercellular signaling (20, 22–24). These diverse and powerful functions of miRNAs lay the foundation for their potential use in clinical diagnosis and treatments of many diseases such as CVDs.

MiR-223-3p is recognized as the guide strand of the miR-223 duplex and used to be called miR-223 (25). Although the other strand named miR-223-5p or miR-223* has been recognized functional in some cases recently (26, 27), most studies have been focused on the regulatory effects of miR-223-3p. MiR-223-3p is initially thought to be a myeloid-specific miRNA affecting hematopoiesis, immune response, and inflammatory diseases (28–31), but currently has been reported to express in many other types of cells such as hepatocytes and cardiomyocytes (32, 33). It has been demonstrated that miR-223-3p is related to multiple pathological processes or features of CVDs including atherosclerosis, vascular or myocardium remodeling, abnormal platelet reactivity, and myocardium ischemia (33–37), which draws our attention to the potential role of miR-223-3p in the diagnosis and treatments of CVDs. In this review, we will systemically elaborate the role of miR-223-3p in different pathophysiological activities causing CVDs and highlight the function of miR-223-3p as therapeutic target, as well as diagnostic or predictive biomarker in these diseases.

## MiR-223-3p in Lipid Metabolism Dysfunction and Atherosclerosis

Lipid metabolism dysfunction, represented by disturbed triglyceride and cholesterol homeostasis, as well as abnormal level and function of lipoproteins, may lead to severe lipid-associated diseases, including atherosclerosis, non-alcoholic fatty liver disease, and diabetes. Atherosclerosis is thought to be a driving force for coronary artery disease and ischemic cerebral stroke, mediated by complex mechanisms including lipid metabolism disorder, vascular cellular dysfunction, chronic inflammation, and plaque development (8). Cholesterol biosynthesis, transport, and efflux are essential for maintaining systemic lipid homeostasis and a high level of plasma cholesterol has been considered a risk factor for atherosclerosis development (38). Lipoproteins, especially the atheroprotective high-density lipoproteins (HDLs) and the atherogenic low-density lipoproteins, carry on different roles in cholesterol metabolism and atherosclerosis (39, 40). MiR-223-3p has been involved in modulating cholesterol homeostasis and transport function of lipoproteins and also has been proved to regulate cellular activities contributing to the pathogenesis of atherosclerosis.

### Effect of MiR-223-3p on Lipoprotein Function and Cholesterol Metabolism

Lipoproteins can combine with nucleic acids to form complex and transport nucleic acids systemically or specifically (41–43). HDLs are responsible for reversing the transport of cholesterol from periphery back to liver and finally excreting cholesterol out of the body (39). As an important member of lipoproteins especially in atherosclerosis, HDLs are capable of delivering endogenous miRNAs including miR-223-3p to neighbor cells for intercellular communication and cellular target genes regulation, and this delivery ability may present differently in pathological situations such as hypercholesterolemia (24, 44, 45). MiR-223-3p was demonstrated as one of the most abundant plasma HDL-miRNAs in familial hypercholesterolemia patients or atherosclerotic mice, and also quite abundant in HDLs of healthy subjects (24). HDL-carried miR-223-3p which generated from polymorphonuclear neutrophils (PMNs) and macrophages was upregulated by native HDLs through increasing the expression of Dicer, and the export of miR-223-3p from PMNs and macrophages to HDLs was increased by HDLs and PMNs activation through promoting the activity of protein kinase C (PKC) and production of reactive oxygen species (ROS) (45). Excessive dietary intake of trans-fatty acids, as one of the risk factors of CVDs, also has been reported to have an impact on HDL-carried miR-223-3p through affecting the plasma HDL-C levels and inflammatory markers (44).

Both native and reconstituted HDLs are capable of acquiring endogenous or exogenous miR-223-3p and delivering it to recipient cells including hepatic and endothelial cells through combination with scavenger receptor class B type I (SR-BI) (24, 46). SR-BI, which is found expressed in hepatocytes, endothelial cells or macrophages, is the primary and high-affinity HDL receptor, and thus plays an important role in regulating cholesterol homeostasis (47, 48). There is an interaction between SR-BI and miR-223-3p. It was demonstrated that SR-BI could facilitate the selective uptake of HDL-cholesterol (HDL-C) and HDL-carried miR-223-3p by cells, as well as might affect miR-223-3p levels in PMNs and macrophages (45), while miR-223-3p, in turn, decreased the expression of SR-BI in hepatocytes, resulting in reduced HDL-C uptake (32, 49). Besides, miR-223-3p also regulates other proteins involved in cholesterol metabolism to reduce cholesterol biosynthesis and promote cholesterol efflux.

MiR-223-3p is abundant in human liver and primary hepatocytes (50). Hepatic miR-223-3p is either delivered from plasma into the liver or natively expressed in hepatocytes. The abundance and functional activity of miR-223-3p increased with elevation of cholesterol levels in hepatocytes and circulation (32). Hepatic miR-223-3p decreased the mRNA and protein level of SR-BI through directly targeting the binding sites of 3′-untranslated region in SR-BI mRNA of human while inhibition of endogenous miR-223-3p abrogated these effects, proving that SR-BI was a downstream target of miR-223-3p in human. This posttranscriptional regulation of SR-BI markedly reduced the uptake of HDL-C and lipid accumulation in hepatic cells (32, 49). The similar regulatory effects of miR-223-3p on SR-BI were also found in human coronary arterial endothelial cells, suggesting the endothelial protection by miR-223-3p. In addition to SR-BI, two cholesterol biosynthetic enzymes 3-hydroxy-3-methylglutaryl-CoA synthase 1 (HMGCS1) and methylsterol monooxygenase 1 (SC4MOL) were found to be direct targets of miR-223-3p, resulting in cholesterol biosynthesis repression by miR-223-3p. Moreover, miR-223-3p also increased efflux of cholesterol by upregulating ATP-binding cassette transporter A1 (ABCA1) through directly targeting transcription factor Sp3 (32). Thus, the reduced uptake of HDL-C and biosynthesis of cholesterol, as well as the promoted efflux of cholesterol, are likely to decrease the liver cholesterol levels. These regulatory mechanisms involved in human cholesterol metabolism by miR-223-3p imply that miR-223-3p may influence plasma lipid levels and lipid-related diseases such as hypercholesterolemia or atherosclerosis in a diverse manner. Based on the experiments conducted in miR-223(-3p/5p) knockout mice, the depletion of miR-223(-3p/5p) contributed to hypercholesterolemia and the elevation of plasma cholesterol levels were associated with HDL-C. However, the elevated HDL-C levels caused by miR-223(-3p/5p) deficiency were thought to be the results of different regulation of target genes in rodents such as SR-BI, which was found inhibited in miR-223(-3p/5p) deficient mice (32). Therefore, the role of miR-223-3p on plasma lipid levels and lipid-related diseases in human still needs further researches.

**Figure 1:**
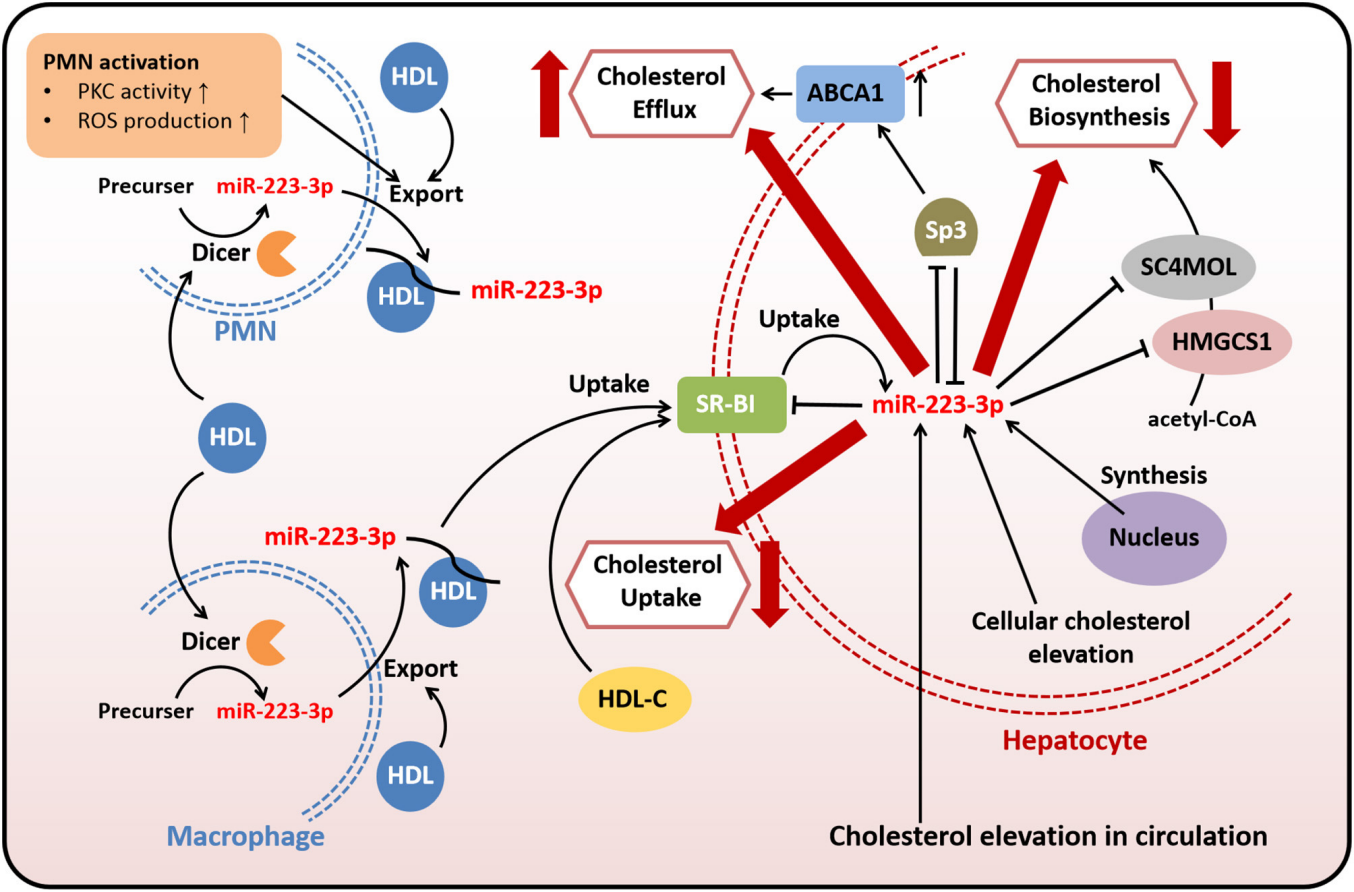
MiR-223-3p is involved in cholesterol transport, biosynthesis, and efflux. Both endogenous and exogenous miR-223-3p in hepatocytes contribute to maintaining cholesterol homeostasis through targeting multiple genes related to cholesterol transport, biosynthesis, and efflux. Exogenous miR-223-3p is mostly originated from PMNs or macrophages and transported by HDLs. The uptake of HDL-carried miR-223-3p depends on SR-BI which is identified as one of the targets of miR-223-3p. Other targets involved in cholesterol metabolism include HMGCS1, SC4MOL and Sp3. MiR-223-3p directly inhibits the expression of these proteins to reduce the cholesterol uptake and biosynthesis, as well as to increase the cholesterol efflux. MiR-223-3p levels in hepatocytes can be upregulated with the elevation of cellular or circulating cholesterol.

Taken together, miR-223-3p modulates cholesterol metabolism through directly or indirectly targeting genes related to cholesterol transport, biosynthesis, and efflux (Figure 1). Both HDL-carried miR-223-3p and hepatocyte-expressed miR-223-3p could execute the regulatory function. MiR-223-3p could inhibit the expression of SR-BI, while the inhibition could lead to reduced uptake of HDL-carried miR-223-3p from plasma, so this loop regulation may be one of the mechanisms helping to maintain the cholesterol homeostasis in cellular or systemic level.

### Effect of MiR-223-3p on Vascular Cells, Platelets, and Macrophages

Vascular endothelial cells (VECs) and vascular smooth muscle cells (VSMCs), as well as platelets and macrophages, are involved in all stages of atherosclerosis and play vital roles in plaque development (8). Endothelial dysfunction contributes to cellular inflammatory phenotypes through activating multiple signaling pathways and increasing the excretion of pro-inflammatory factors, consequently prompting monocytes recruitment and differentiation into macrophages which could form inflammatory foam cells in the lesions of vessel walls (51, 52). Aberrant proliferation, differentiation, and migration of VSMCs promote atherosclerosis progression through the formation of neointima and plaque (53). MiR-223-3p is firstly described in hematopoietic cells and blood cells and recent studies have shown that miR-223-3p is a pleiotropic miRNA in several tissues (28, 34). Although some studies consider that miR-223-3p may be barely expressed in VECs or VSMCs, a considerable amount of miR-223-3p which probably derived and excreted from platelets, monocytes, or macrophages has been found in these two types of cells, executing anti-inflammatory and anti-atherosclerotic effects (35, 46, 54). In addition, miR-223-3p impacts atherosclerosis also via regulating cytopoiesis, cell survival, and the function of platelets and macrophages (55–57).

VECs can ingest HDL-carried miR-223-3p or miR-223-3p from activated-platelet-derived exosomes in plasma (46, 54). Platelet activation displays pro-atherosclerotic effects, resulting from its role in endothelial damages and thrombus formation, however, activated-platelet-derived exosomes are found to be protective for atherosclerosis and endothelial inflammation (54, 58). The components contained in activated-platelet-derived exosomes included a high level of miR-223-3p, which was found upregulated in exosomes of atherosclerotic models (54). MiR-223-3p ingested by VECs contributed to the downregulation of intercellular adhesion molecule 1 (ICAM1), which could be upregulated by tumor necrosis factor α (TNF-α) or thrombosis (46, 54). Given that miR-223-3p was thought to be barely expressed in VECs and mostly from extracellular sources, it indicates that extracellular miR-223-3p is capable of regulating gene expression in cells. MiR-223-3p can suppress the inflammation and endothelial activation through directly or indirectly regulating ICAM1 in VECs. MiR-223-3p downregulated the expression of ICAM1 through directly targeting or depending on the suppression of nuclear factor kappa B (NFκB) and mitogen-activated protein kinase (MAPK) signaling pathways, with inhibiting cytoplasm-to-nucleus translocation of NFκB p65 and phosphorylation of p38, c-Jun N-terminal kinase (JNK), and extracellular signal-regulated kinase (ERK) (46, 54). MiR-223-3p could also be transcribed and secreted from platelets and leukocytes to serum, and then entered VSMCs and vascular walls, resulting in elevation of miR-223-3p levels in serum and vascular walls of atherosclerotic animals and patients. MiR-223-3p reduced VSMCs proliferation and migration, as well as increased cell apoptosis, through directly targeting insulin-like growth factor 1 receptor (IGF1R) to inhibit its downstream phosphatidylinositol 3-kinase (PI3K)/protein kinase B (AKT) pathway with reduced phosphorylation of AKT. The regulation on these signaling pathways affected cellular activities of VSMCs, and ultimately suppressed vascular neointimal formation and atherosclerosis (35). Although these findings have been argued by other studies of miR-223-3p on VSMCs function, which indicated that overexpression of miR-223-3p promoted the proliferation and migration of VSMCs (59) and thus downregulation of miR-223-3p might decrease neointimal hyperplasia and prevent restenosis after angioplasty through augmenting expression of genes implicated in VSMCs differentiation and contractility (60), it is quite evident that miR-223-3p can help to prevent pathological changes in atherosclerosis.

Platelets and macrophages play vital roles in the development of plaque and thrombosis during the atherosclerotic progression. As one of the most abundant miRNAs in platelets (61–63), miR-223-3p showed a mild acceleration on platelets production but with no influence on platelets function (55). It suggests that platelet function may not depend on regulation pathways mediated by miR-223-3p. Contrast to effects on platelets, miR-223-3p plays significant roles in macrophages differentiation, survival, and function during inflammatory stimulation and atherosclerosis. As the same as activated platelets, macrophages generate microvesicles containing miRNAs (64). Although miR-223-3p contained in activated macrophage-derived microvesicles could be transported to monocytes and induce the differentiation from monocytes to macrophage, as well as macrophages survival, suggesting the pro-inflammatory effects of microvesicles containing miR-223-3p (56), the upregulation of miR-223-3p in macrophages presented protective effects against atherosclerosis through decreasing macrophage foam cells, macrophage-mediated lipid deposition, and excretion of pro-inflammatory factors. The anti-atherosclerotic effects of miR-223-3p in macrophages were performed through activating the PI3K/AKT pathway with upregulation of p-AKT to inhibit the toll-like receptor 4 (TLR4)/NFκB pathway with suppression of phosphor-p65. The expression of miR-223-3p was reduced in lipopolysaccharide-induced macrophages, while in lesions of atherosclerotic animals it was upregulated, probably in a feedback way (57).

**Figure 2:**
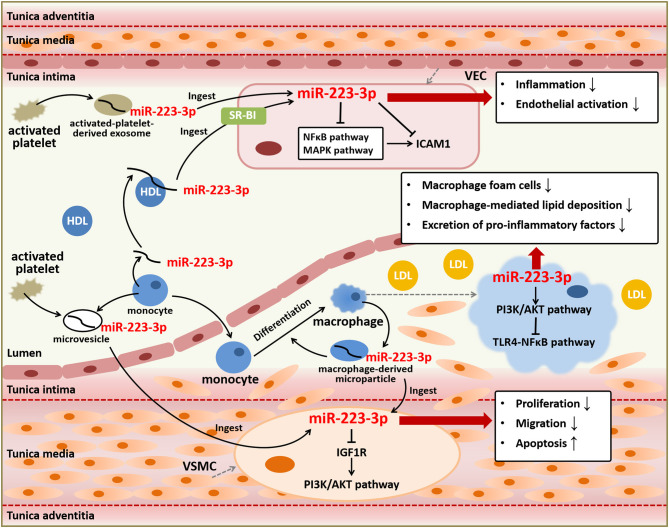
Anti-atherosclerotic role of miR-223-3p. MiR-223-3p plays important roles in atherosclerotic progression based on its effects on various types of cells involved in atherosclerosis including VECs, VSMCs, and macrophages. MiR-223-3p can suppress endothelial inflammation and activation through targeting ICAM1 and regulating NFκB and MAPK signaling pathways. MiR-223-3p also alleviates vascular remodeling mediated by proliferation and migration of VSMCs and lipid deposition and inflammation mediated by macrophages through PI3K/AKT or TLR4/NFκB signaling pathways. Besides, MiR-223-3p is an important endocrine signal factor for communication between platelets, monocytes, and vascular cells.

To summarize, miR-223-3p can function as an endocrine genetic signal for intercellular communication among blood cells, immune cells, and vascular cells, mediated by HDLs, platelet-derived exosomes, or macrophage-derived microparticles. It also plays essential roles against atherosclerotic progression primarily through anti-inflammation, endothelial protection, reducing vascular remodeling, and decreasing lipid deposition (Figure 2), indicating its therapeutic role for atherosclerosis and related CVDs.

### MiR-223-3p as a Potential Biomarker for Atherosclerosis

The characteristics of miRNAs, which can be easily acquired from plasma or serum in patients and conveniently detected by commonly used techniques, make it an important potential biomarker in multiple diseases (8, 65). Circulating levels of miR-223-3p have been associated with atherosclerotic diseases and hyperlipidemia (35, 66–68). It was found that miR-223-3p levels were elevated in serum and atherosclerotic vessels of patients with atherosclerosis, as well as atherosclerotic models established in apolipoprotein E deficient mice (35). Hypertension associated with hyperlipidemia, as the major risk factor leading to atherosclerotic diseases, could mimic the atherosclerotic situation and increase the plasma and platelet levels of miR-223-3p but reduced the levels in platelet-derived microvesicles in hamsters (68). However, other research indicated that plasma miR-223-3p levels were reduced in patients with abdominal aortic aneurysm, an atherosclerosis-related disease (66). The lower level of plasma miR-223-3p was correlated with increased atherosclerotic manifestations including coronary artery disease, peripheral arterial disease and stroke in heart failure patients, and elevated levels of atherosclerosis-related indicators such as growth differentiation factor 15, as well as augmented risk of cardiovascular-related rehospitalization (67). Thus, patients or animal models with only atherosclerosis or atherosclerotic situation seem to manifest a higher level of circulating miR-223-3p, while in patients combined with other diseases, atherosclerosis is associated with reduced circulating miR-223-3p. Although miR-223-3p has the potential to protect against atherosclerosis based on its regulation of lipid metabolism and cellular activities involved in atherosclerosis, its role to function as a biomarker of atherosclerosis diagnosis or risk prediction remains indeterminate. Further researches to investigate the explicit role of miR-223-3p as a diagnostic or prognostic biomarker for atherosclerosis and atherosclerosis-related diseases based on large cohorts and rigorous designs are needed.

## MiR-223-3p in Coronary Artery Disease

Coronary artery disease is usually originated from atherosclerotic pathological alterations which lead to coronary artery narrow or block and ultimately ischemia of myocardium (69). Therefore, it is also known as coronary heart disease or ischemic heart disease, including multiple clinical manifestations such as stable angina pectoris and acute coronary syndrome (ACS), and affects lots of people worldwide (69, 70). There have been many researches uncovering the effects of miR-223-3p in coronary artery disease, mostly in regard to its adverse impacts on myocardial infarction and potential regulatory mechanisms involved in antiplatelet therapy, as well as its role in diagnosis and risk prediction of coronary artery disease.

### MiR-223-3p in Myocardial Infarction

Myocardial infarction, as a main category of ACS, is a worldwide devastating coronary artery disease with high morbidity and mortality, usually caused by a severe reduction of blood flow to the cardiac tissues and the accompanying myocardial ischemia or hypoxia (7). Cardiac damages induced by these conditions are closely related to processes such as cardiomyocytes apoptosis, oxidative stress, and inflammation, so therapies to alleviate these processes, as well as angiogenic factors, are conducive to recover cardiac function (7, 71). Pathological conditions present during or after myocardial infarction include myocardial fibrosis, arrhythmia, and ischemic/reperfusion (I/R) injury, etc (7, 72–74). Some researches have indicated that miR-223-3p can protect cardiomyocytes from myocardial I/R-induced inflammation and necroptosis, while others have proved that miR-223-3p displays pro-apoptosis and anti-angiogenesis effects, as well as promotes myocardial fibrosis and arrhythmia induced by myocardial infarction.

I/R injury, commonly induced by interventional therapies such as percutaneous coronary intervention or thrombolytic therapy, which are necessary when myocardial infarction occurred, could result in cardiomyocytes apoptosis and necrosis and has been demonstrated to be manipulated by gene regulation (26, 75, 76). MiR-223-3p has been reported upregulated in I/R hearts and involved in mechanisms underlying I/R-induced cardiomyocytes necroptosis in myocardial infarction (26, 77). MiR-223-3p cooperative with miR-223-5p inhibited cell necroptosis involved in I/R-induced myocardium injuries through regulating inflammatory response and necrotic signaling pathway respectively, which together promoted the necroptosis (26, 78–80). MiR-223-3p could attenuate I/R-triggered inflammation through suppressing the NOD-like receptor family pyrin domain containing 3 (NLRP3) inflammasome signaling pathway. MiR-223-3p directly targeted IkB kinase α (IKKα) and NLRP3 to abrogate the activation of NLRP3 inflammasome and downregulate the expression of TNF-α and interleukin 1 β (IL-1β), the inflammatory cytokines involved in I/R-triggered inflammation. While miR-223-5p repressed the necrotic signaling pathway through the inhibition of the necrosome containing receptor-interacting protein 3 (RIP3) with its partners receptor-interacting protein 1 (RIP1) and mixed lineage kinase domain-like (MLKL) (26). Taken together, miR-223-3p may exert beneficial effects on I/R-induced inflammatory response and cardiomyocytes necroptosis following myocardial infarction and other ischemic heart diseases.

Despite the protective effects on I/R-induced damages, more studies have regarded miR-223-3p as a potential therapeutic target that should be suppressed in myocardial infarction due to its effects of pro-apoptosis in cardiomyocytes and antiangiogenesis. Angiogenesis, which is normally restricted by the physiological activity of VECs, is essential for the recovery of myocardial ischemia and myocardial infarction. It was demonstrated that miR-223-3p was highly expressed in natively quiescent VECs which were freshly isolated from human vessels, while in VECs that were cultured for angiogenesis with stimulation of growth factors the miR-223-3p were downregulated to very low levels (81). This sharp downregulation of miR-223-3p is in accordance with previous studies that suggested nearly undetectable expression of miR-223-3p in cultured VECs and may result from the epigenetic silencing mechanisms or a lack of exogenous miR-223-3p delivered by other cells from plasma *in vivo* as we mentioned in the former parts (46, 54, 81). MiR-223-3p in VECs reduced cellular proliferation and migration to inhibit angiogenesis through targeting integrin β 1 (ITGB1). Downregulation of ITGB1 suppressed the phosphorylation of vascular endothelial growth factor receptor 2 (VEGFR2) and fibroblast growth factor receptor 1 (FGFR1) of growth factor (GF) signaling pathway, leading to inhibition of AKT signaling pathway, while the antagonist of miR-223-3p reversed these antiangiogenic effects (81). Another study revealed that miR-223-3p was upregulated in ischemia cardiac microvascular endothelial cells from a myocardial infarction model and also played antiangiogenic roles. MiR-223-3p inhibited endothelial cells proliferation and migration through targeting ribosomal protein S6 kinase B1 (RPS6KB1) to inhibit hypoxia inducible factor 1 α (HIF-1α) signaling pathway, an important pathway for angiogenesis, and affect the downstream proteins including vascular endothelial growth factor (VEGF), MAPK, PI3K, and AKT (37).

Furthermore, miR-223-3p was found to be concerned with the hypoxia-induced cardiomyocytes apoptosis. Hypoxia is a common condition during myocardial ischemia and acts as a promoting factor of apoptosis, contributing to ischemic heart diseases such as myocardial infarction (82, 83). It was proved that miR-223-3p in cultured cardiomyocytes was increased under 1% O_2_ hypoxia and directly suppressed Kruppel-like factor 15 (KLF15), a regulator of cardiomyocyte structure and function to repress cardiac hypertrophy, to promote hypoxia-induced apoptosis and oxidative stress of cardiomyocytes (82), although others reported that 10% O_2_ alveolar hypoxia induced reduction of miR-223-3p expression in ventricular myocardium of mice (84). Inhibition of miR-223-3p reduced hypoxia-induced cardiomyocytes apoptosis with the induction of anti-apoptotic protein B cell lymphoma 2 (BCL2) and reduction of pro-apoptotic protein B cell lymphoma 2-associated X (BAX), as well as decrease of cleaved caspase 3. Besides, inhibition of miR-223-3p also repressed the production of ROS and decreased peroxidation activities under hypoxia with reducing the malondialdehyde (MDA) contents and enhancing antioxidant enzymes such as superoxide dismutase (SOD), catalase (CAT), and glutathione peroxidase 1 (GPX1), to alleviate hypoxia-induced oxidative stress (82).

Myocardial fibrosis is a pathological change that could occur after myocardial infarction to obstruct the renewal of myocardium, mainly resulting from abnormal proliferation and differentiation of cardiac fibroblasts which take place of dead cardiomyocytes, and finally leads to myocardial remodeling and stiffness, as well as other complications related to myocardial infarction (85–88). MiR-223-3p has been shown to participate in fibrosis, including the cardiac fibrosis after myocardial infarction (85, 89). MiR-223-3p, which could be regulated by transforming growth factor β (TGF-β), was abundant in activated cardiac fibroblasts than cardiomyocytes and could stimulate cardiac fibroblasts proliferation, migration, differentiation, collagen synthesis, and α-smooth muscle actin (ACTA2) expression. The pro-fibrosis effects mediated by miR-223-3p were through targeting RAS p21 protein activator (RASA1), a RAS signal negative regulator, to upregulate the downstream MAPK and PI3K/AKT signaling pathways with increasing the phosphorylation of MAPK kinase 1/2 (MEK1/2), ERK1/2, and AKT, leading to myocardial fibrosis and deterioration of ventricular function (85).

Arrhythmia is another severe disorder following myocardial infarction and caused by electrophysiological dysfunction, especially characterized by extended action potential due to the reduction of transient outward K^+^ current (I_to_) (90–92). MiR-223-3p was thought to have the potential to regulate potassium voltage-gated channel subfamily D member 2 (KCND2), a subunit of voltage-gated transient outward K^+^ channel carrying I_to_, probably leading to a reduction of I_to_ (93). A recent study verified this and proved the role of miR-223-3p suppressing arrhythmia in myocardial infarction. In the ventricular cardiomyocytes of a myocardial infarction animal model, it was found that miR-223-3p levels were elevated, while expression of KCND2 and I_to_ were diminished resulting from the direct inhibition of KCND2 by miR-223-3p, and the upregulation of miR-223-3p promoted arrhythmia following myocardial infarction (94). Therefore, blocking the endogenous miR-223-3p is possible to develop into a therapeutic method for the prevention of myocardial fibrosis and arrhythmia after myocardial infarction or other ischemia situations, consequently improving the prognosis of these diseases.

**Figure 3:**
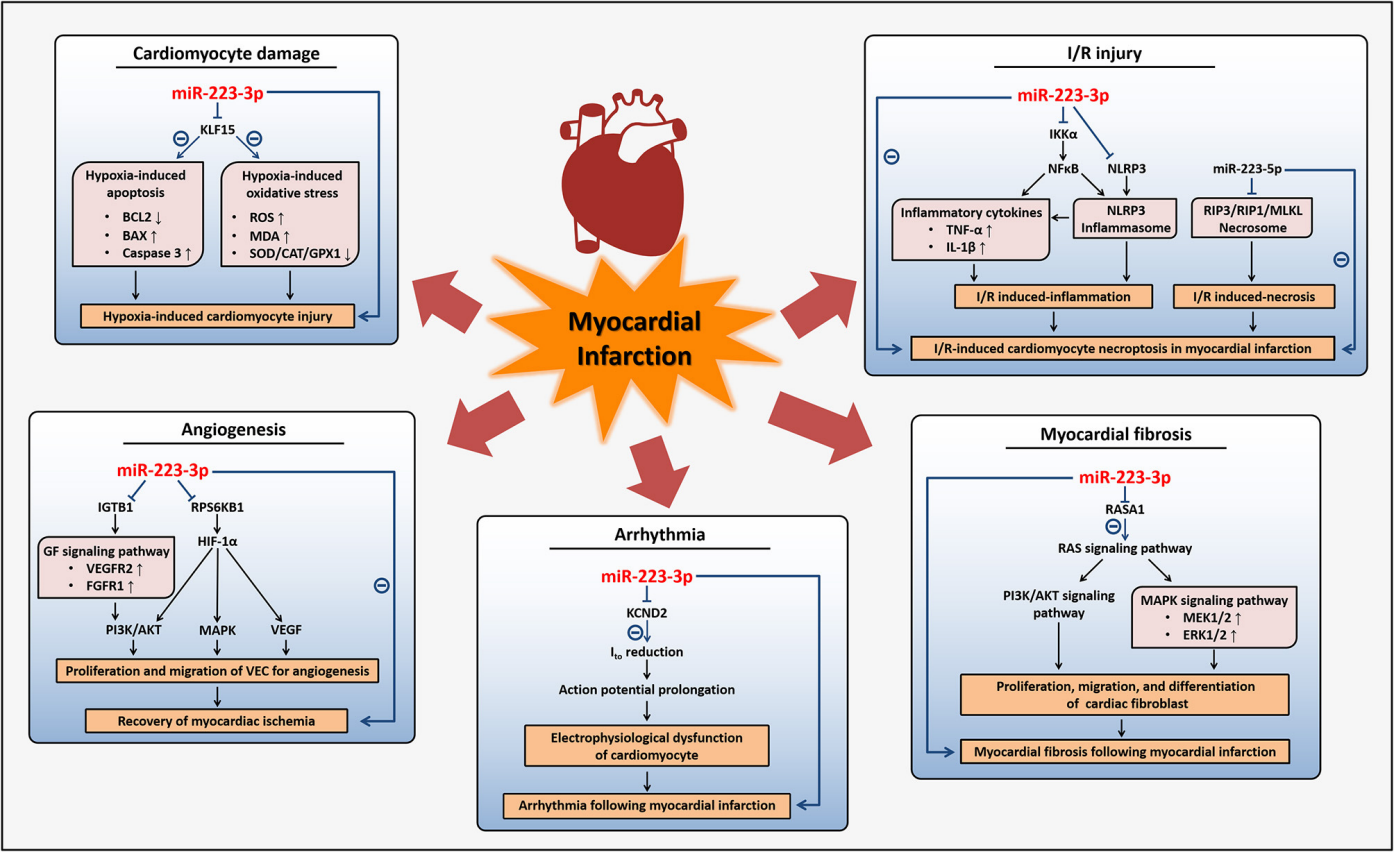
Regulatory mechanisms of miR-223-3p in myocardial infarction. MiR-223-3p participates in different pathophysiological activities affecting myocardial infarction through regulation on multiple genes and diverse signaling pathways. MiR-223-3p promotes hypoxia-induced cardiomyocyte injury and inhibits the recovery of myocardial ischemia with its anti-angiogenesis effects during myocardial infarction. Myocardial fibrosis and arrhythmia can be induced by miR-223-3p to impact the prognosis of myocardial infarction. Besides, miR-223-3p is helpful for alleviation of I/R injury which often occurs after myocardial infarction.

Therefore, on the one hand, miR-223-3p may act as an inducer of myocardial infarction due to its promotion of hypoxia-induced cardiomyocytes apoptosis, antagonism to angiogenesis, and induction of complications following myocardial infarction including fibrosis and arrhythmia. On the other hand, miR-223-3p may improve I/R injury after myocardial infarction based on its inhibition of inflammatory response and necroptosis in cardiomyocytes (Figure 3). Although the expression of miR-223-3p was found increased in cardiomyocytes of myocardial infarction models and heart tissues of patients with myocardial infarction (94, 95), the study investigating the potential of circulating miR-223-3p as a biomarker related to myocardial infarction remains scarce.

### MiR-223-3p as a Potential Biomarker and Therapeutic Target for Coronary Artery Disease

Although at present there exist some efficient medical interventions for the treatment of coronary artery disease such as percutaneous coronary intervention, clinical non-invasive biomarkers, which could accurately and conveniently be used for assisting diagnosis, assessing the severity, evaluating the efficacy of treatment, and predicting prognosis of coronary artery disease, are still in need. Based on the multiple functions of miR-223-3p associated with the pathophysiological processes of coronary artery disease, it is expected to become a biomarker for the disease.

#### Role of MiR-223-3p in Antiplatelet Therapy

Platelet activity contributes to the pathophysiological process in coronary artery disease, especially the formation of thrombosis (96). Antiplatelet therapy plays a significant role in pharmacological prevention of cardiovascular events and markedly impacts the prognosis of coronary artery disease, including the classic dual antiplatelet therapy (DAPT) with aspirin and clopidogrel, combination of aspirin and other P2Y12 antagonists, or monotherapy with one single antiplatelet drug (97). Responsiveness to the antiplatelet drugs and the platelet activity in patients with ischemic CVDs determine the benefits from antiplatelet therapy, while poor responsiveness or high platelet activity, known as high on-treatment platelet reactivity, is related to the risk of adverse cardiovascular outcomes (98–101). MiR-223-3p is quite abundant in both platelets and plasma, and directly targets P2Y12 in platelets, making it important for antiplatelet drugs, especially the P2Y12 antagonists used in antiplatelet therapy such as clopidogrel and ticagrelor (35, 62, 102, 103).

In patients with non-ST elevation ACS who received a regular DAPT with aspirin and clopidogrel, it was indicated that both the levels of miR-223-3p in plasma and platelets were downregulated in low clopidogrel response patients (104, 105), characterized by the high platelet reactivity index (PRI) of patients based on the measurement of vasodilator-stimulated phosphoprotein phosphorylation (106–108). MiR-223-3p levels were negatively correlated with PRI and the reduced miR-223-3p in plasma or platelets was demonstrated to be the only independent predictive factor of low responders to clopidogrel determined by PRI, compared with other factors that could potentially affect platelet reactivity, such as age, genotypes, use of calcium channel blockers or proton-pump inhibitors, and diabetes (104, 105). Therefore, low levels of circulating miR-223-3p may be a promising biomarker to predict the poor responsiveness to clopidogrel in ACS patients and the decrease of miR-223-3p in platelets is probably involved in the mechanism of clopidogrel resistance based on its inhibition of P2Y12 in platelets.

While downregulation of miR-223-3p might increase the platelet reactivity to reduce the efficacy of DAPT containing clopidogrel, the enhanced inhibition of platelet reactivity by more potent P2Y12 antagonists ticagrelor or prasugrel instead of clopidogrel of DAPT in patients with non-ST elevation ACS was associated with the elevation of plasma miR-223-3p (109). Therefore, miR-223-3p levels seem to be positively correlated with the effectiveness of antiplatelet therapy with P2Y12 antagonists. Besides, it was reported that in patients with ACS, plasma miR-223-3p level decreased 24 h after switching the DAPT from clopidogrel to ticagrelor in order to further inhibit platelet reactivity (96). Another study also showed that inhibition of platelet reactivity in healthy volunteers with prasugrel or DAPT containing prasugrel decreased the circulating miR-223-3p (62). In patients with coronary artery disease who received antiplatelet treatment for 24 h, the severity of coronary artery disease based on SYNTAX score was positively correlated with the platelet level of miR-223-3p, which might indirectly reflect the relation between platelet reactivity and miR-223-3p (110). Thus, although miR-223-3p can inhibit P2Y12 in platelets to affect the platelet reactivity, the circulating miR-223-3p levels may also be influenced by degree of P2Y12 inhibition and associated with platelet reactivity, and as a result, the definite correlation between circulating miR-223-3p levels and platelet reactivity might depend on status of patients, categories of drugs, or administration time. Even so, circulating miR-223-3p is helpful to predict the efficacy of antiplatelet therapy, especially the responsiveness to clopidogrel in ACS patients. Thus, circulating miR-223-3p may be useful for identifying patients suitable to receive clopidogrel as one of the antiplatelet drugs, thus saving the time and cost of switching therapeutic strategy for these patients.

#### Role of MiR-223-3p in Diagnosis, Severity Assessment, and Risk Prediction of Coronary Artery Disease

So far, the effective and convenient biomarker for coronary artery disease is still lacking. It was reported that in patients with coronary artery disease, the circulating miR-223-3p levels were elevated and positively associated with the severity of coronary atherosclerotic lesions evaluating by Gensini scores (111). The increased circulating miR-223-3p in coronary artery disease also could be used to predict cardiovascular death risk for patients, in particular for patients with ACS (112). Besides, compared with patients with stable coronary artery disease, the plasma HDL-carried miR-223-3p in ACS patients decreased during the transcoronary passage, indicating that in ACS patients the uptake of HDL-carried miR-223-3p by the coronary vascular wall was promoted, which suggested the positive correlation between uptake of plasma HDL-carried miR-223-3p and severity of coronary artery disease (113). Thus, miR-223-3p may probably be developed into a biomarker for assessing the severity and predicting the death risk of coronary artery disease. The levels of serum miR-223-3p were upregulated in myocardial infarction patients, compared with healthy controls and angina pectoris patients (114). While another study showed that circulating miR-223-3p levels were negatively correlated to the risk of myocardial infarction (102). More researches are needed to confirm the role of miR-223-3p as a diagnostic or predictive biomarker of myocardial infarction. Furthermore, the expression of miR-223-3p in peripheral blood mononuclear cells was downregulated in patients who were diagnosed as coronary artery disease with significant or insignificant stenosis compared with healthy controls, which was most prominent in patients with insignificant stenosis, indicating that miR-223-3p in peripheral blood mononuclear cells might be positively related to severity of coronary artery disease and could be used for discriminate patients with significant or insignificant stenosis (115).

Taken together, owing to the complex mechanisms and diverse symptoms of coronary artery disease, miR-223-3p may play multiple roles in these diseases, depending on its regulatory effects on the development of atherosclerosis, myocardial ischemia or hypoxia, and platelet function. The circulating miR-223-3p can be developed into both a therapeutic target and a biomarker to evaluate the treatment effects in coronary artery disease because it is capable of regulating the platelet aggregation and predicting the responsiveness to antiplatelet drugs of patients. MiR-223-3p is a promising biomarker that can be used for evaluating the severity of coronary artery disease such as stenotic degree of coronary atherosclerosis and predicting the prognosis of coronary artery disease, for its convenient detection, lower cost, and less damage to human body. Future studies should be more focused on the specific diagnostic or predictive function of miR-223-3p in coronary artery disease of different categories, or with different pathogenic mechanisms and inducing factors.

## MiR-223-3p in Heart Failure

Heart failure is one of the most serious CVDs because of its high morbidity and mortality, mainly characterized by exhausted cardiac function of systole and diastole, and threatens worldwide people for a lack of effective treatments (116, 117). Cardiac hypertrophy, especially pathological cardiac hypertrophy, which acts as a cardiac compensatory mechanism in response to multiple pathophysiological stress, leads to cardiac ventricle remodeling and is an important pathogenic factor for heart failure (118). Other cardiac ventricle remodeling processes including fibrosis and malignant arrhythmia also contribute to the development of heart failure (119–121). Besides, pro-inflammatory conditions in myocardium that may result from systemic inflammation, such as activation and polarization of macrophages infiltrating into the myocardium, also promote heart failure progression (122, 123). Researchers have found numerous heart failure-related miRNAs such as miR-223-3p which prominently plays a part in heart failure through regulating cardiomyocytes hypertrophy and inflammation. In addition, miR-223-3p levels have been proved closely related to complications or combined symptoms of heart failure.

### Effect of MiR-223-3p on Pathophysiological Processes in Heart Failure

Current researches have demonstrated that miR-223-3p has biphasic effects on heart failure through bidirectional regulation of cardiac hypertrophy and promotion of inflammatory response. Both in the rat model of cardiac hypertrophy induced by transverse aorta constriction and in hypertrophic cardiomyocyte models treated with different inducing reagents endothelin-1 or phenylephrine, the expression of miR-223-3p in myocardium and cardiomyocytes were downregulated (124, 125). The mechanism underlying the phenylephrine-induced downregulation of miR-223-3p in cardiomyocytes was suppressing glycogen synthase kinase 3 β activity to reduce β-catenin degradation and increase its activation (125). This modulation upregulated SRY-box transcription factor 2, which could promote cardiomyocytes proliferation and increase heart size, to inhibit the transcription of primary miR-223 (125, 126). Inhibition of primary miR-223 increased the stromal interaction molecule 1 (STIM1), a target of miR-223-3p, to induce cardiomyocyte hypertrophy through elevating the cytosolic Ca^2+^ levels of cardiomyocytes (125, 127–129). When overexpressing miR-223-3p or miR-223(-3p/5p) in cardiomyocytes, the hypertrophy was ameliorated mainly manifesting as smaller cellular surface area, downregulation of classic pathological hypertrophy marker genes encoding atrial natriuretic peptide (ANP), B type natriuretic peptide (BNP), α-actinin, and myosin heavy chain 6/7 (MYH6/7) (124, 125). The protective effects of miR-223-3p against cardiomyocytes hypertrophy might also depend on direct inhibition of troponin I3 interacting kinase (TNNI3K), a cardiac kinase that was upregulated in hypertrophic cardiomyocytes and induced cardiomyocytes hypertrophy through regulating cardiac remodeling process (124, 130, 131). Overexpression of miR-223-3p decreased the phosphorylation of cardiac troponin I, the downstream target of TNNI3K, and reversed the increased intracellular Ca^2+^ concentration and contractility of cardiomyocytes that were induced by TNNI3K, thus resulting in amelioration of cardiomyocytes hypertrophy (124). Notably, TNNI3K is also thought to be a promoter of physiological cardiac hypertrophy (132), so miR-223-3p may target TNNI3K to suppress either physiological or pathological cardiac hypertrophy.

Similar to the findings in models of cardiac hypertrophy, miR-223-3p was also downregulated in the myocardium of patients with non-end stage heart failure, more prominently in non-diabetic heart failure patients compared with diabetic heart failure patients or healthy controls (133). Among the advanced heart failure patients who received heart transplantation directly or after a period of left ventricular assist devices support, the expression of miR-223-3p in failing hearts before transplantation was significantly higher in patients with a period of left ventricular assist devices support and positively correlated with the cardiac index values, which reflect the cardiac output and function. This suggested that miR-223-3p might be involved in cardiac reverse remodeling and heart recovery (134). Therefore, miR-223-3p can be downregulated in models of cardiac hypertrophy or heart failure and might make a beneficial contribution to heart failure primarily through alleviating the ventricle remodeling processes concerned in cardiac hypertrophy, promoting the heart recovery process, and improving the cardiac function.

However, others have reported that overexpressed miR-223-3p could induce both pathological and physiological cardiac hypertrophy, as well as regulate the inflammatory response in hearts, probably resulting in the development of heart failure. MiR-223-3p was found upregulated in hearts of end stage heart failure patients and mouse models of cardiac hypertrophy induced by isoproterenol treatment or transverse aorta constriction (33, 135, 136), suggesting the detrimental roles of miR-223-3p in cardiac hypertrophy and heart failure, which was opposite to the above-mentioned studies (124, 125, 133). Both pathological and physiological cardiac hypertrophy have been found in transgenic mouse models with cardiac-specifically overexpression of miR-223(-3p/5p) (33, 137). These transgenic mice could develop pathological cardiac hypertrophy at the age of 8–12 weeks, characterized by higher heart/body weights, larger size of cardiomyocytes, interstitial fibrosis, upregulation of pathological cardiac hypertrophy marker genes in hearts, and impaired cardiac function which could lead to heart failure. Furthermore, in miR-223(-3p/5p) knockout mice, isoproterenol-induced pathological cardiac hypertrophy and heart failure were alleviated, also indicating the harmful role of miR-223(-3p/5p) in heart failure (33). MiR-223-3p directly targeted apoptosis repressor with CARD (ARC), which was reduced in human failing hearts and involved in cardiomyocytes hypertrophy and apoptosis, to induce pathological cardiac hypertrophy in animals and cardiomyocytes (33, 138, 139). Aiming to block the inductive effects of miR-223-3p for cardiac hypertrophy and heart failure, researchers screened a heart-related circular RNA to directly bind miR-223-3p and increase the expression of ARC of cardiomyocytes, resulting in alleviation of cardiac hypertrophy and heart failure induced by isoproterenol (33). This throws light on the development of the promising therapeutic target for heart failure.

The opposite effects of miR-223-3p in cardiac hypertrophy progression and heart failure may result from different stimuli and species to establish models of cardiac hypertrophy, considering different mechanisms of cardiac hypertrophy inducers such as endothelin-1, phenylephrine, or isoproterenol, and the species variation such as rats, mice, and human. The different expressions of miR-223-3p among patients may be due to diverse progression of disease such as non-end stage or end stage of heart failure. Besides, in experiments overexpressing miR-223-3p *in vitro* or *in vivo*, different regulatory effects on cardiac hypertrophy were observed, as *in vitro* studies showed inhibition on cardiac hypertrophy while *in vivo* studies showed induction on cardiac hypertrophy (33, 124, 125, 137). It is believed that *in vivo* studies are closer to real pathophysiological processes in cardiac hypertrophy and heart failure in human. Therefore, it is reasoned that miR-223-3p may display an inductive effect on cardiac hypertrophy and heart failure in human body, although more studies are needed.

Physiological cardiac hypertrophy is an adaptive response of hearts which is often caused by exercise training and characterized by relatively normal cardiac structure and function without fibrosis or abnormal expression of pathological cardiac hypertrophy gene markers such as ANP, BNP, and MYH7 (140, 141). Physiological cardiac hypertrophy was found in 3, 6, or 12-months aged transgenic mice overexpressing miR-223(-3p/5p), only manifesting larger hearts and thicker left ventricular walls with enhanced cardiac function (137). The inductive effects of miR-223(-3p/5p) on physiological cardiac hypertrophy were mediated by activation of the AKT signal pathway which played significant roles in the promotion of physiological cardiac hypertrophy (137, 142, 143). MiR-223-3p repressed the degradation of HIF-1α through directly inhibiting F-Box and WD repeat domain containing 7 (FBXW7) or targeting activin A receptor type 2A (ACVR2A) to downregulate egl-9 family hypoxia inducible factor 3 (EGLN3). The activated HIF-1α signaling pathway could increase phosphorylation of AKT directly or through upregulating periostin. Meanwhile, miR-223-3p in cardiomycytes was found to directly target two upstream positive regulators of AKT pathway, IGF1R and ITGB1, tending to inhibit the phosphorylation of AKT. Although both positive and negative regulators of AKT were influenced, the overexpression of miR-223(-3p/5p) led to the activation of AKT signaling pathway and induced physiological cardiac hypertrophy in models (137). Dual contribution of miR-223-3p overexpression to both pathological and physiological cardiac hypertrophy might result from the difference of transgenic models construction, as the length of DNA fragments containing primary miR-223 and the sequences of the primers used to generate transgenic models were different (33, 137), possibly leading to the diverse abundance of miR-223-3p overexpression and effects on cardiac hypertrophy.

In addition, miR-223-3p could also accelerate heart failure depending on its pro-inflammatory effects in macrophages including activation and polarization of macrophage toward a pro-inflammatory (M1) phenotype, as well as induction of inflammation markers and secretion of cytokines, probably contributing to the development of heart failure (122, 123, 144).

Conclusively, miR-223-3p regulates pathophysiological processes that drive heart failure, including hypertrophy of cardiomyocytes and myocardium, as well as the inflammatory response. Although the specific role of miR-223-3p in cardiac hypertrophy and heart failure remains controversial, the therapeutic method to repress miR-223-3p with a heart-related circular RNA and thus to alleviate cardiac hypertrophy and heart failure has been identified (33). Therefore, miR-223-3p seems to be a promising therapeutic target in clinical treatments of heart failure.

### MiR-223-3p as a Potential Biomarker for Heart Failure

Circulating biomarkers can facilitate the diagnosis, therapy, and prognostic assessment of heart failure. Currently, the classic and recommended biomarkers for heart failure evaluation only refer to BNP or N terminal pro BNP (NT-proBNP) (145). However, BNP or NT-proBNP is not a specific biomarker for heart failure because some other diseases also display an elevation of BNP or NT-proBNP, such as cardiopulmonary disease and renal failure. Thus, to screen more effective and specific biomarkers for heart failure is still in need. Based on the stability and functional diversity, miRNAs could be potential and powerful biomarkers for heart failure (146, 147). The relationship between circulating miR-223-3p and heart failure has been investigated both in human and animal models.

There are evidences that in acute heart failure patients, plasma miR-223-3p decreased significantly compared with healthy controls or chronic heart failure patients, and its further decline was associated with the increased risk of 180-day mortality in these patients (148, 149). In addition, the plasma miR-223-3p level decreased 48 h after hospitalization, and its level was negatively correlated with circulating growth differentiation factor 15 that was identified as one of the parameters for worse clinical outcomes in acute heart failure. The correlation was more obvious in the severest group of patients, while not observed at the time of admission (150). The circulating levels of miR-223-3p in heart failure patients were also negatively related to rehospitalization caused by CVDs (67). Both atherosclerotic diseases and renal injuries are common in heart failure (67, 151). It was reported that in heart failure patients the reduced circulating miR-223-3p was associated with increased manifestations of atherosclerosis and elevation of atherosclerosis-related indicators (67). Circulating levels of miR-223-3p were also negatively associated with the tubular damage biomarker neutrophil gelatinase associated lipocalin, which could signify early worsening of renal function in heart failure (152–154). These concomitant situations often aggravate the severity of heart failure and lead to adverse prognosis in patients, so looking for biomarkers, including the candidate miR-223-3p, to precisely predict the progression and risk, even comorbidities or complications of the disease is of great value in the treatment of heart failure.

Whereas, in muscular dystrophy patients combined with advanced heart failure and a hypertension-induced congestive heart failure rat model, plasma miR-223-3p levels were elevated and positively correlated with circulating level of heart failure marker BNP and cardiac MYH7 expression in the animal model, which were upregulated in heart failure (155–159). It is noteworthy that there exist differences in circulating miRNAs signature between rodent heart failure models and human. The observed alteration of circulating miR-223-3p levels in heart failure patients did not occur in multiple rodent models with well-established heart failure (160). MiR-223-3p was identified to have similar sequences between human and mice, but different in rats. The diversity of miRNAs signature of different species may also due to the limitation of experimental animal models that could usually only mimic the heart failure symptoms caused by one stimulus but not accurately and roundly replicate the diverse pathogenesis of clinical human heart failure, and heart failure animal models are more likely to approximate the state of chronic heart failure in human. Furthermore, etiological factors in heart failure, such as hypertension, ischemia, and myocardial dystrophy, should also be considered when investigating the role of biomarkers related to heart failure.

Taken together, circulating levels of miR-223-3p are associated with the severity, current biomarkers for worse clinical outcomes, and the mortality of heart failure, as well as the risk of rehospitalization and concomitant diseases. Thus, circulating miR-223-3p level might not only evaluate the progression of heart failure but also act as a prognostic indicator or a predictor for comorbidities and complications of heart failure. Also, based on the regulatory networks of miR-223-3p in processes involved in heart failure, it is more likely to be a potential therapeutic target and serve as a biomarker to evaluate the treatment effects of heart failure.

## MiR-223-3p in Other CVDs

### MiR-223-3p in Pulmonary Arterial Hypertension

Pulmonary arterial hypertension (PAH) is a serious and progressive disease commonly characterized by pulmonary arterial obstruction and elevated pulmonary arterial pressure (161). These conditions are commonly caused by excessive pulmonary vasoconstriction, inflammation, and pulmonary vascular remodeling, and are usually accompanied by hypoxic condition and dysfunction of pulmonary vasculature, leading to right ventricular failure or even death (161–163). As one of the most significant mechanisms involved in vascular remodeling of PAH, aberrant proliferation, migration, and apoptosis of pulmonary arterial endothelial cells and pulmonary arterial smooth muscle cells (PASMCs) affect the progression of PAH (164–166). Recent studies have verified the regulatory effects of miR-223-3p on PAH and PAH-related dysfunction of PASMCs.

MiR-223-3p was downregulated in lung, pulmonary arteries and PASMCs of both PAH patients and PAH models (167–169). In serum of female PAH patients associated with congenital heart disease, miR-223-3p levels were reduced compared with healthy females while there was no difference in males, suggesting the potential of miR-223-3p as a biomarker for PAH associated with congenital heart disease in females (168). Researchers have explored the specific mechanisms underlying the regulatory effects of miR-223-3p on PAH. HIF-1α, which is usually upregulated under hypoxia, was activated in PAH and led to the inhibition of miR-223-3p in PASMCs of PAH patients, resulting in upregulation of multiple miR-223-3p targets and the development of PAH (167–169). MiR-223-3p directly targeted integrin β 3 (ITGB3), IGF1R, poly (ADP-ribose) polymerase 1 (PARP1), ras homolog family member B (RHOB), and myosin light chain 2 (MYL2) in PASMCs to repress the proliferation and migration, and to promote the differentiation and DNA damage-induced apoptosis (167–172). Besides, the downregulation of miR-223-3p and upregulation of ITGB3 both increased the expression of IGF1R and PARP1, indicating that miR-223-3p could suppress IGF1R and PARP1 through direct regulation or by targeting ITGB3 (169). Therefore, both overexpression of miR-223-3p or suppression of ITGB3 regulated PASMCs activities to alleviate the pathological features of PAH animals including pulmonary vascular remodeling, pulmonary or myocardial hemorrhage, fibrosis of lung or myocardium, and right ventricular hypertrophy. The reverse of PAH phenotypes reduced the pulmonary resistance, improved the cardiac function, and finally prolonged the survival time of animals (167–169). Taken together, miR-223-3p inhibits PAH through directly targeting multiple genes and signaling pathways involved in abnormal activities of PASMCs and pulmonary vascular remodeling, so it might be a promising therapeutic target for PAH.

### MiR-223-3p in Inflammation-Related Myocardial Disease

MiR-223-3p is considered to play a key role in inflammatory or immune responses during varieties of diseases through targeting different genes involved in monocytes differentiation, macrophages activation, and polarization, as well as regulation of eosinophil cells, resulting in modulation of both pro-inflammatory and anti-inflammatory signaling pathways (25, 30, 173). Myocarditis is a kind of inflammatory heart disease usually caused by infectious factors including virus and bacteria while non-infectious factors may also trigger the immune response in myocardium, such as autoimmune myocarditis (174, 175). Myocarditis has become one of the most common reasons for sudden death in children and young people (174, 176). It was reported that miR-223-3p displayed a protective function on both coxsackievirus B3 (CVB3)-induced viral myocarditis and autoimmune myocarditis, as well as on inflammation-related myocardial depression in polymicrobial sepsis (27, 177, 178).

In heart tissues and macrophages that infiltrated in hearts of CVB3-infected mice, the expression of miR-223-3p was downregulated, accompanied by elevation of PBX/knotted 1 homeobox 1 (PKNOX1). Upregulation of miR-223-3p altered the polarization phenotypes of macrophages from classical pro-inflammatory M1 type to alternative anti-inflammatory M2 type, partly depending on directly targeting PKNOX1 (177). This transition manifested as the inhibition of M1 markers such as nitric oxide synthase 2 and TNF-α, as well as the increase of M2 markers such as arginase-1 and fizz-1 (177, 179). These effects of miR-223-3p on macrophage activation and polarization made contributions to alleviating CVB3-induced inflammation and myocarditis with reducing pro-inflammatory and myocardial injury markers including interferon-γ, interleukin 6 (IL-6), creatine kinase MB, lactate dehydrogenase, and aspartate transaminase, as well as increasing the anti-inflammatory factor interleukin 10. As a result, miR-223-3p improved the indexes of cardiac function, body weights, and survival of CVB3-infected animals, indicating the alleviation of CVB3-induced myocarditis (177, 180, 181).

The protective role of miR-223-3p in autoimmune myocarditis lies in the regulation of another kind of immune cells, dendritic cells (DCs) which have been recognized as essential regulators to balance immunity and tolerance depending on their activation status (178, 182, 183). The tolerogenic DCs (tDCs) can be induced by immunosuppressive factors including interleukin 10 and TGF-β, leading to the unresponsiveness of T cells and tolerance of inflammatory stimulation, which is helpful in autoimmune diseases (184, 185). MiR-223-3p levels were reduced in serum and DCs of experimental autoimmune myocarditis mice while upregulating miR-223-3p in DCs prompted their transition to tDCs with reduced cellular surface markers of mature DCs and increased anti-inflammatory cytokines. MiR-223-3p also alleviated autoimmune myocarditis *in vivo* through reducing pericardial inflammation, increasing immunosuppressive response, and improving cardiac function. MiR-223-3p induced the transition of DCs to tDCs through directly targeting NLRP3, an important regulator to maintain immune homeostasis and tolerance, resulting in reduced production of NLRP3 inflammasome and its downstream signaling molecules such as caspase-1 and IL-1β (178).

In addition to myocarditis, miR-223-3p could also alleviate the myocardial depression in polymicrobial sepsis cooperated with miR-223-5p (27). Myocardial depression induced by sepsis is a major cause of cardiac dysfunction and septic cardiomyopathy, as well as increases the mortality in sepsis (186, 187). Both miR-223-3p and miR-223-5p were found downregulated in the polymicrobial sepsis model, and deficiency of miR-223(-3p/5p) weakened cardiac systolic function and reduced survival rate of animals in sepsis model. MiR-223(-3p/5p) deficiency also aggravated the inflammation induced by severe sepsis in myocardium, peritoneal fluid, and blood, with elevation of pro-inflammatory factors including TNF-α, IL-6, and IL-1β, increased bacterial load in body fluids, and neutrophil infiltration in hearts (27). The sepsis-induced myocardial depression can be inhibited via different pathways by miR-223-3p and miR-223-5p. MiR-223-3p targeted both signal transducer and activator of transcription 3 (STAT3) and IL-6 to inhibit the pro-inflammatory signaling pathway while miR-223-5p targeted semaphorin 3A to improve the autonomic control of cardiac function (27, 188). These effects of miR-223(-3p/5p) reduced the inflammation of heart and alleviated cardiac dysfunction, resulting in amelioration of myocardial depression and reduction of sudden death in severe sepsis (27, 189–191).

### MiR-223-3p in Hypertension

Hypertension is one of the most common diseases and has become a major risk factor for many other diseases, such as myocardial infarction, stroke, and chronic renal failure (192–195). To overcome the clinical challenges of adverse effects of drugs and failure to achieve the optimum blood pressure control in hypertension, it requires earlier detection and accurate therapy to manage blood pressure and reduce its complications (196, 197). MiRNAs have been considered as useful biomarkers or indicators in many diseases depending on their properties such as high stability, easy detection, and low cost, to predict and evaluate the progression or risk of diseases as above-mentioned. Studies have illuminated the role of miRNAs in diagnosis and treatment of hypertension (198–200). A few researches have indicated that miR-223-3p might also be a promising biomarker for the diagnosis of hypertension and prediction of CVDs risk in hypertensive patients (201, 202).

Circulating miR-223-3p levels were downregulated in hypertensive patients, and the reduced serum miR-223-3p along with miR-199a-3p, miR-208a-3p, and miR-122-5p exhibited better diagnostic effects on hypertension than other miRNAs, with the highest sensitivity (202). Another research attempt to uncover the role of platelet miR-223-3p in hypertension and its related risk of CVDs. Platelets, which are abundant with miRNAs, are involved in the progression of hypertension-related pro-thrombotic status and blood pressure elevation during thrombogenesis (201, 203, 204). To explore the miRNAs in platelets could be helpful to screen biomarkers for hypertension. Platelet miR-223-3p levels decreased in hypertensive patients and the levels were negatively correlated with the systolic blood pressure of patients. Besides, the platelet levels of miR-223-3p in hypertensive patients were lower in patients with other CVDs, so platelet miR-223-3p could be a prognostic biomarker for hypertension (201). Therefore, both the circulating and platelet miR-223-3p might be developed into biomarkers for diagnosis and prognosis estimation of hypertension.

### MiR-223-3p in Arrhythmia

Arrhythmia is an important category of CVDs and may occur alone or with other diseases. Severe or frequent arrhythmia attack could lead to hemodynamic disorder, heart failure, and sudden death. MiR-223-3p expression was increased in atrial tissues of atrial fibrillation (AF) patients with rheumatic heart disease, as well as in animal models of AF, suggesting the possible role of miR-223-3p involved in pathogenesis of AF (205). As we mentioned in previous parts, miR-223-3p induced arrhythmia following myocardial infarction through decreasing KCND2 and I_to_, indicating the potential of miR-223-3p to regulate electrophysiological functions of myocardium (94). More effects of miR-223-3p in arrhythmia are worth investigating.

## Conclusion

CVDs remain a major public health problem and result in heavy burden for human society. We have summarized the role of miR-223-3p in CVDs and recent developments in its potential as biomarker and therapeutic target for CVDs. MiR-223-3p is widely expressed in varieties of cell types that link to CVDs including monocytes, macrophages, platelets, hepatocytes, endothelial cells (resting state), cardiomyocytes, and cardiac fibroblasts to influence their cellular activities. These regulatory effects primarily depend on suppressing the mRNAs of target genes involved in lipid metabolism, inflammation, cell proliferation or apoptosis, electrophysiology, cell hypertrophy, and cell polarization (Table 1). Besides, MiR-223-3p participates in regulatory networks of multiple signaling pathways such as NFκB, MAPK, PI3K/AKT, RPS6KB1/HIF-1α, and NLRP3 inflammasome pathways to play diverse roles in CVDs (Table 2). Nevertheless, there still exist controversies about the effects of miR-223-3p in some CVDs. Studies to determine the specific role of circulating miR-223-3p for diagnosis and prediction of various CVDs are further needed. Future works to modulate the local level of miR-223-3p may lead to more effective treatment of CVDs.

**Table 1:** Verified target genes of miR-223-3p in CVDs.

**Gene**	**Gene function**	**Cell/Tissue type**	**Species**	**References**
*SR-BI*	Cholesterol transport	Hepatocyte	Human	(32, 49)
		VEC		(32)
HMGCS1^*^	Cholesterol biosynthesis	Hepatocyte	Human	(49)
SC4MOL^*^	Cholesterol biosynthesis	Hepatocyte	Human	(49)
Sp3^*^	Cholesterol efflux	Hepatocyte	Human	(49)
ICAM1^*^	Inflammation	VEC	Human	(46)
*IGF1R*	Proliferation, migration, apoptosis	VSMC	Rat, mouse	(35)
	Proliferation, differentiation	PASMC	Human, rat, mouse	(169, 170)
	Hypertrophy	Cardiomyocyte	Rat, mouse	(137)
*NLRP3*	Inflammation, necroptosis	Cardiomycyte	Rat, mouse	(26)
	Inflammation	Dendritic cell	Mouse	(178)
IKKα^*^	Inflammation, necroptosis	Cardiomycyte	Rat, mouse	(26)
*ITGB1*	Proliferation, migration	VEC	Human, mouse	(81)
	Hypertrophy	Cardiomyocyte	Rat, mouse	(137)
RPS6KB1^*^	Proliferation, migration	VEC	Rat	(37)
KLF15^*^	Hypoxia-induced apoptosis, oxidative stress	Cardiomycyte	Rat	(82)
RASA1^*^	Proliferation, migration, differentiation	Cardiac fibroblast	Rat	(85)
KCND2^*^	Electrophysiology	Cardiomyocyte	Rat	(94)
*P2Y12*	Platelet aggregation, thrombus growth/stability	Platelet	Human	(103)
		Megakaryocyte		
STIM1^*^	Hypertrophy	Cardiomyocyte	Rat	(125)
TNNI3K^*^	Hypertrophy	Cardiomyocyte	Rat	(124)
ARC^*^	Hypertrophy	Cardiomyocyte	Mouse	(33)
FBXW7^*^	Hypertrophy	Cardiomyocyte	Rat, mouse	(137)
ACVR2A^*^	Hypertrophy	Cardiomyocyte	Rat, mouse	(137)
ITGB3^*^	Proliferation, differentiation, DNA damage-induced apoptosis	PASMC	Rat	(169)
PARP1^*^	DNA damage-induced apoptosis	PASMC	Human, rat	(167, 169)
RHOB^*^	Proliferation, migration	PASMC	Human, rat	(168)
MYL2^*^	Proliferation, migration	PASMC	Human, rat	(168)
PKNOX1^*^	Activation, polarization	Macrophage	Mouse	(177)
*STAT3*	Inflammation	Heart	Mouse	(27)
*IL-6*	Inflammation	Heart	Mouse	(27)

* *The target genes marked with an asterisk are cell-specific targets of miR-223-3p corresponding to the certain cell types in the table according to the current research*.

**Table 2:** Signaling pathways regulated by miR-223-3p in CVDs.

**Signaling pathway**	**Cell function**	**Disease/Process**	**References**
NFκB	Inflammation of VEC	Atherosclerosis	(54)
	Lipid accumulation and inflammation of macrophage	Atherosclerosis	(57)
	Inflammation and necroptosis of cardiomycyte	Myocardial I/R injury	(26)
TLR4	Lipid accumulation and inflammation of macrophage	Atherosclerosis	(57)
MAPK	Proliferation, migration, and inflammation of VEC	Atherosclerosis, cardiac angiogenesis	(37, 54)
	Proliferation, migration, and differentiation of cardiac fibroblast	Myocardial fibrosis following myocardial infarction	(85)
PI3K/AKT	Proliferation, migration, and apoptosis of VSMC	Atherosclerosis	(35)
	Inflammation of macrophage	Atherosclerosis	(57)
	Proliferation and migration of VEC	Cardiac angiogenesis	(37, 81)
	Hypertrophy of cardiomyocyte	Cardiac hypertrophy	(137)
	Proliferation, migration, and differentiation of cardiac fibroblast	Myocardial fibrosis following myocardial infarction	(85)
IGF1	Proliferation, migration, and apoptosis of VSMC	Atherosclerosis	(35)
	Hypertrophy of cardiomyocyte	Cardiac hypertrophy	(137)
	Proliferation and differentiation of PASMC	PAH	(169, 170)
NLRP3 inflammasome	Inflammation and necroptosis of cardiomycyte	Myocardial I/R injury	(26)
	Inflammation of dendritic cell	Autoimmune myocarditis	(178)
HIF-1α	Hypertrophy of cardiomyocyte	Cardiac hypertrophy	(137)
	Proliferation and migration of VEC	Cardiac angiogenesis	(37)
GF	Proliferation and migration of VEC	Cardiac angiogenesis	(37, 81)
BCL2 family-mediated apoptosis	Hypoxia-induced apoptosis of cardiomyocyte	Myocardial hypoxia	(82)
IL-6/STAT3	Inflammation of myocardium	Septic cardiomyopathy	(27)
RAS	Proliferation, migration, and differentiation of cardiac fibroblast	Myocardial fibrosis following myocardial infarction	(85)

## Author Contributions

M-WZ: conceptualization and writing—original draft. Y-JS: literature collection. JS: visualization. J-GY: conceptualization and writing—review and editing. All authors contributed to the article and approved the submitted version.

## Conflict of Interest

The authors declare that the research was conducted in the absence of any commercial or financial relationships that could be construed as a potential conflict of interest.
